# A model comparison study of the flowering time regulatory network in *Arabidopsis*

**DOI:** 10.1186/1752-0509-8-15

**Published:** 2014-02-11

**Authors:** Charles CN Wang, Pei-Chun Chang, Ka-Lok Ng, Chun-Ming Chang, Phillip CY Sheu, Jeffrey JP Tsai

**Affiliations:** 1 Department of Biomedical Informatics, Asia University, Taichung, Taiwan; 2 Department of Applied Informatics and Multimedia, Asia University, Taichung, Taiwan; 3 Department of Computer Science, University of Illinois at Chicago, Chicago, USA; 4 Department of Electrical Engineering & Computer Science, University of California at Irvine, Irvine, USA

**Keywords:** Dynamic model, Michaelis-Menten model, Mass-action model, S-system, Arabidopsis, Floral transition process

## Abstract

**Background:**

Several dynamic models of a gene regulatory network of the light-induced floral transition process in *Arabidopsis* have been developed to capture the behavior of gene transcription and infer predictions based on experimental observations. It has been proven that the models can make accurate and novel predictions, which generate testable hypotheses.

Two major issues were addressed in this study. First, construction of dynamic models for gene regulatory networks requires the use of mathematic modeling that comprises equations of a large number of parameters. Second, the binding mechanism of the transcription factor with DNA is another factor that requires detailed modeling. The first issue was tackled by adopting an optimization algorithm, and the second was addressed by comparing the performance of three alternative modeling approaches, namely the S-system, the Michaelis-Menten model and the Mass-action model. The efficiencies of parameter estimation and modeling performance were calculated based on least square error (*O(p)*), mean relative error (MRE) and Akaike Information Criterion (AIC).

**Results:**

We compared three models to describe gene regulation of the flowering transition process in *Arabidopsis*. The Mass-action model is the simplest and has the least parameters. It is therefore less computation-intensive with the smallest AIC value. The disadvantage, however, is that it assumes the system is simply a second order reaction which is not the case in our study. The Michaelis-Menten model also assumes the system is homogeneous and ignores the intracellular protein transport process. The S-system model has the best performance and it does describe the diffusion effects. A disadvantage of the S-system is that it involves the most parameters. The largest AIC value also implies an over-fitting may occur in parameter estimation.

**Conclusions:**

Three dynamic models were adopted to describe the dynamics of the gene regulatory network of the flowering transition process in *Arabidopsis*. Based on MRE*,* the least square error and global sensitivity analysis, the S-system has the best performance. However, the fact that it has the highest AIC suggests an over-fitting may occur in parameter estimation. The result of this study may need to be applied carefully when modeling complex gene regulatory networks.

## Background

*Arabidopsis thaliana* is a plant in the mustard family that has been frequently chosen as the organism model in research on plant science. It possesses small size, diploid genetics, small genome and relatively short generation time. The life cycle of Arabidopsis from vegetative to reproductive growth is an important developmental step that is under tight genetic control. In the meanwhile the floral transition state has shown to be integrated by a complex gene regulatory network.

For *Arabidopsis,* floral organ specification has been successfully linked to spatial gene expression patterns according to floral transition and floral development. This model has five pathways that can explain various external (photoperiod, vernalization, ambient temperature) and internal (autonomous, age, gibberellins) conditions to regulate the floral transition through an elaborated genetic network [1-5].

Recently, gene expression data sets have become available for the genes involved in the regulation of floral transition and flower development in *Arabidopsis*. In [6], time series of gene expression were presented for each class of genes in the floral transition group. For most floral transitions, the majority of which are members of *Arabidopsis,* namely *APETALA* (*AP1*) and *LEAFY* (*LFY*), are transcription factors where the way in which they are activated was known from experiments [7]. Furthermore, it has been shown that in regulating the flowering time in *Arabidopsis,* these two information sources open the door for mathematical model development.

To inference gene regulatory networks from time course data has been one of the main challenges in systems biology. In recent years, technological advances have driven the development of systems biology in experimental methods that generate *in vivo* time course data to characterize regulatory interactions. In the last years there has been a significant increase of publications in the area of model construction. Some examples include: cell fate determination in *Arabidopsis* flowers [8], model study of role proteins (*CLV1*, *CLV2*, *CLV3* and *WUS*) in shoot apical meristem of *Arabidopsis*[9], integration of developmental and hormonal pathways in the *Arabidopsis* flower [10], and gene regulatory network models for plant development [11].

However, a major challenge with such models is that the detailed transcript binding process in a microscopic picture is usually unclear; therefore these models may be deviated from the reality. In addition, a dynamic model requires extensive mathematical formula and large amount of experimental data that are not available. Alternatively, a large-scale gene regulation model can be constructed based on stoichiometry without a large number of fitted parameters. Although these models can be used to predict the regulation behaviour using flux analysis, they failed to capture the transient behaviors of genes. For instance, Mahadevan [12] proposed the dynamic flux balance analysis for situations where there is knowledge available; Yugi [13] proposed a method that aims to simplify the number of kinetic parameters in building a dynamic model.

Many studies on dynamic simulation of gene regulation systems have been reported in the literature. Spieth [14] used linear weight matrices, S-system and H-systems model, and different optimization algorithms to model a nonlinear dynamic system. Rafael et al. [15] compared Michaelis-Menten model, power-law and generalized mass action to represent an *E. coil* central carbon metabolic network.

In this study, we modeled the regulatory interactions in the flowering of *Arabidopsis* with a series of kinetic functions. The first case considers the conditions that mRNA is produced immediately after transcript factor binding. This process is formulated as a mass action model. The second case assumes that a complex state is formed between the transcription factor and its target gene. The production of mRNA is delayed due to the stability of the complex state. This process is formulated as a Michaelis-Menten model. The third case assumes that the binding process of the transcript factor is limited by 1-D and 3-D diffusions, and the production of mRNA is dominated by a diffusion-reaction process. Accordingly S-system was adopted in this study to model such a mechanism.

## Results

### Comparison of the models

Table 1 presents the reaction mechanisms depicted in the flowering time regulatory network of *Arabidopsis*. This gene regulatory network describes the flowering time (Photoperiodic) in *Arabidopsis thaliana*. The core of this regulatory network includes:

1 The photoperiod activates the *CO* gene.

2 After *CO* activates the expression of *FLOWERING LOCUST (FT)* probably by binding to the *FT* regulatory regions and the *bZIP* transcription factor *FD,* the *FT/FD* complex activates the expression of *SOC1.*

3 *SOC1* and *AGL24* form a positive feedback loop and up-regulate *LFY*.

4 *AP1* and *LFY* are ultimately resolved in the up-regulation of the floral meristem identity genes.

**Table 1:** **A dynamic model for the concentration of gene complexes of the flowering time regulatory network of *Arabidopsis *** **according to the reaction scheme depicted in Figure **4

	**Variable**	**Symbol**	**Description**	**Reference**
Independent				
	X8	FD	Basic-leucine zipper (bZIP) transcription factor family protein	[1]
	X9	PHYB	Phytochrome B	[2]
Dependent				
	X1	CO	Zinc finger protein CONSTANS	[3]
	X2	FT	Protein FLOWERING LOCUS T	[4]
	X4	SOC1	MADS-box protein transcription factor SOC1	[5]
	X5	AP1	Floral homeotic protein APETALA 1	[6]
	X6	AGL24	MADS-box protein AGL24	[7]
	X7	LFY	Protein LEAFY	[8]

In this study, we compared three dynamic models to reconstruct the behaviour of the flowering time regulatory network of *Arabidopsis*. The governing differential equations are listed in the following:

### S-system

$$\begin{array}{l} \overset{˙}{X}1=\alpha_{1}X9^{g19}-\beta_{1}X1^{h11}\\ \overset{˙}{X}2=\alpha_{2}X1^{g21}-\beta_{2}X2^{h22}X8^{h28}\\ \overset{˙}{X}3=\alpha_{3}X2^{g32}X8^{g38}-\beta_{3}X3^{h33}\\ \overset{˙}{X}4=\alpha_{4}X3^{g43}X6^{g46}-\beta_{4}X4^{h44}\\ \overset{˙}{X}5=\alpha_{5}X3^{g53}-\beta_{5}X5^{h55}\\ \overset{˙}{X}6=\alpha_{6}X4g^{64}-\beta_{6}X6^{h66}\\ \overset{˙}{X}7=\alpha_{7}X6^{g76}-\beta_{7}X7^{h77} \end{array}$$

$\overset{˙}{X}8$ and $\overset{˙}{X}9$ are constants.

### Micharlis-Menten model

$$\begin{array}{l} \overset{˙}{X}1=\frac{Vm_{1}\cdot \left[X9\right]}{Km_{1}+\left[X9\right]}-dk_{1}\left[X1\right]\\ \overset{˙}{X}2=\frac{Vm_{2}\cdot \left[X1\right]}{Km_{2}+\left[X1\right]}-dk_{2}\left[X2\right]\\ \overset{˙}{X}3=\frac{Vm_{3}\cdot \left[X2\right]\left[X8\right]}{Km_{3}+\left[X2\right]\left[X8\right]}-dk_{3}\left[X3\right]\\ \overset{˙}{X}4=\left(\frac{Vm_{4a}\cdot \left[X3\right]}{Km_{4a}+\left[X3\right]}\cdot \frac{Vm_{4b}\cdot \left[X6\right]}{Km_{4b}+\left[X6\right]}\right)-dk_{4}\left[X4\right]\\ \overset{˙}{X}5=\frac{Vm_{5}\cdot \left[X3\right]}{Km_{5}+\left[X3\right]}-dk_{5}\left[X5\right]\\ \overset{˙}{X}6=\frac{Vm_{6}\cdot \left[X4\right]}{Km_{6}+\left[X4\right]}-dk_{6}\left[X6\right]\\ \overset{˙}{X}7=\frac{Vm_{7}\cdot \left[X6\right]}{Km_{7}+\left[X6\right]}-dk_{7}\left[X7\right] \end{array}$$

$\overset{˙}{X}8$ and $\overset{˙}{X}9$ are constants.

### Mass-action model

$$\begin{array}{l} \overset{˙}{X}1=kr_{1}\left[X9\right]-dk_{1}[X1]\\ \overset{˙}{X}2=kr_{2}\left[X1\right]-dk_{2}[X2]\\ \overset{˙}{X}3=kr_{3}\left[X2\right][X8]-dk_{3}[X3]\\ \overset{˙}{X}4=\left(\mathrm{kr}{\left[X3\right]}_{4a}\cdot \mathrm{kr}{\left[X6\right]}_{4b}\right)-dk_{4}\left[X4\right]\\ \overset{˙}{X}5=kr_{5}\left[X3\right]-dk_{5}[X5]\\ \overset{˙}{X}6=kr_{6}\left[X4\right]-dk_{6}[X6]\\ \overset{˙}{X}7=kr_{7}\left[X6\right]-dk_{7}[X7] \end{array}$$

$\overset{˙}{X}8$ and $\overset{˙}{X}9$ are constants.

Table 2 summarizes the total number of parameters used in each model. Due to the complex nature of the S-system model, 31 parameters were used to describe the floral transition pathway, whereas the Micharlis-Menten model and the Mass action model used 23 and 15 parameters, respectively. Among them the S-system used the most parameters because the reaction rate was described by a non-linear function for the reactant concentration.

**Table 2:** The total number of parameters in each of the three models used in this study

	**Model**		
	S-system	Mass action	Michaelis-Menten
Total number of parameters	31	15	23

**Figure 1:**
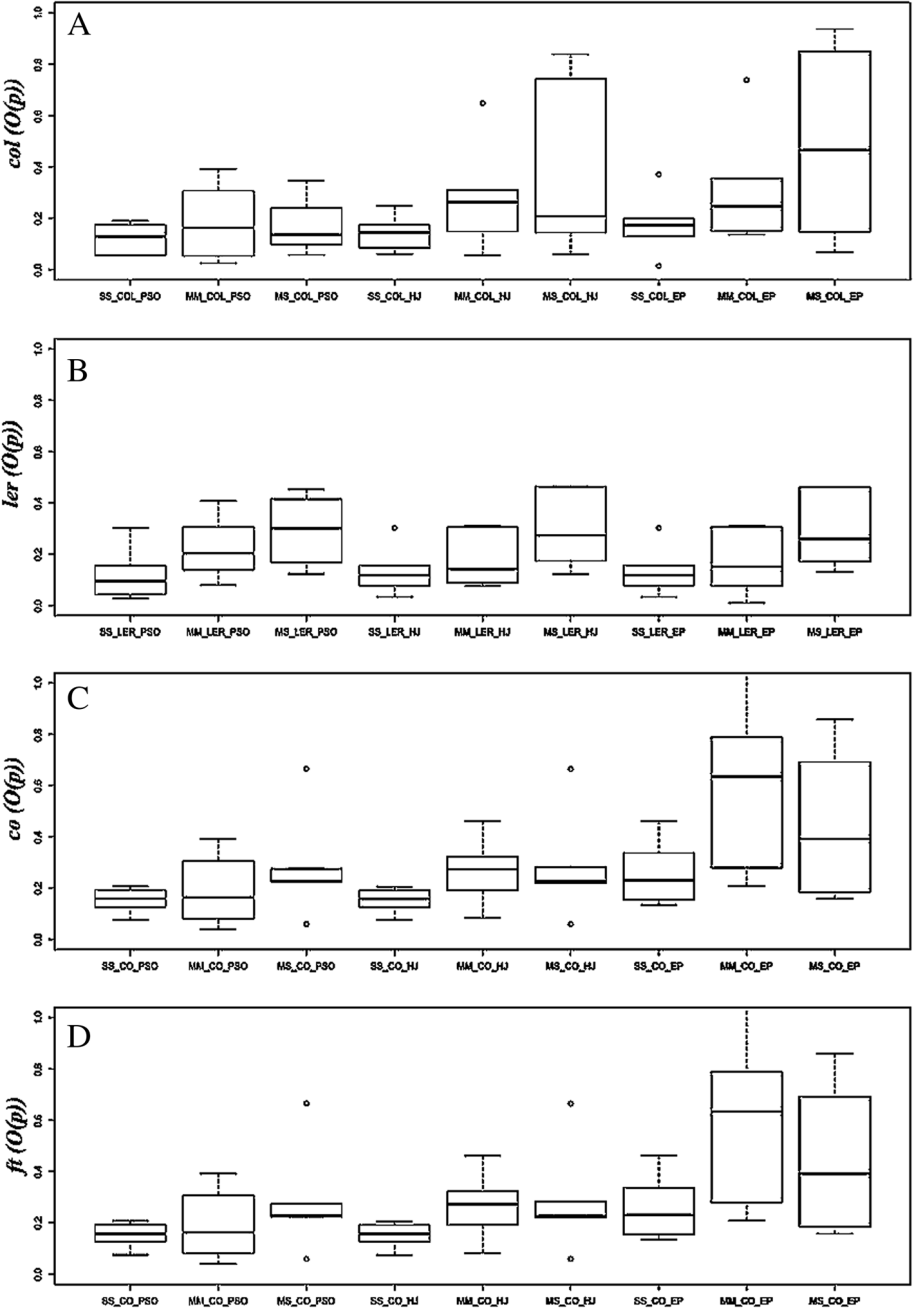
**An analysis of the *O(p) *** **calculated for the three models and three optimization methods in four experimental data sets: (A) *O(p) *** **calculated for the *col *** **experimental data; (B) *O(p) *** **calculated for the *ler *** **experimental data; (C) *O(p) *** **calculated for the *co *** **experimental data; and (D) *O(p) *** **calculated for the *ft *** **experimental data.**

Parameter estimation has been considered as a reverse engineering problem, which may be performed based on local optimization methods and global optimization methods. In this study, three different optimization methods were employed including local HJ (local optimization), EP and PSO (global optimization). The *O(p)* and MRE were used to measure the quality of the fit for the estimated parameters. The values of *O(p)*calculated for the three models and the three optimization methods with four experimental data sets are shown in Figure 1. The result suggests that the PSO method was most suitable for our dynamic models.

**Figure 2:**
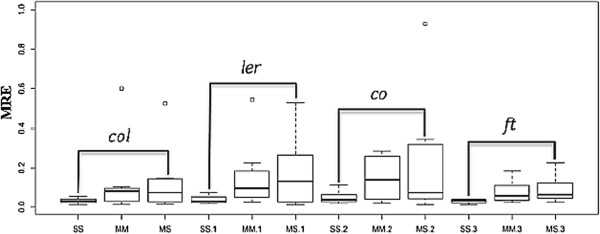
**The *MRE *** **calculated for the three models with four experimental data sets.**

The values of the MRE calculated for the three models and the four experimental data sets are presented in Table 3. The result suggests that the S-system model could achieve the best performance compared with the other two models, as the S-system has the smallest mean relative error (shown in Figure 2).

**Table 3:** The means and standard deviations of MRE calculated for the S-system, Michaelis-Menten model and Mass-action model

**Model**	**col**	**ler**	**co**	**ft**
S-System	0.0325 ± 0.0131	0.0380 ± 0.0193	0.0490 ± 0.0297	0.0312 ± 0.0109
Michaelis-Menten model	0.1294 ± 0.1801	0.1542 ± 0.1595	0.2746 ± 0.3954	0.0775 ± 0.0517
Mass action model	0.1295 ± 0.1570	0.1738 ± 0.1654	0.2266 ± 0.2900	0.0899 ± 0.0628

The AIC calculated for the three models, namely the S-system (*col*, 53.0331; *ler*, 52.6223; *co*, 46.2319; *ft*, 49.6211), Michaelis-Menten model (*col*, 30.1816; *ler*, 38.1605; *co*, 24.6298; *ft*, 34.1275) and Mass-action model (*col*, 26.1364; *ler*, 26.5598; *co*, 10.8465; *ft*, 22.8427), are presented in Table 4. The result suggests that among the three, the Mass-action model is the simplest and has the least parameters. It is therefore less complex with the smallest AIC value.

**Table 4:** The Akaike Information Criterion (AIC) calculated for the S-system, Michaelis-Menten model and Mass action model

**Model**	**col**	**ler**	**co**	**ft**
S-system	53.0331	52.6223	46.2319	49.6211
Michaelis-Menten model	30.1816	38.1605	24.6298	34.175
Mass action model	26.1364	26.5598	10.8465	22.8427

These results suggest that the S-system model represents an option to simulate the dynamics of our gene regulatory network. It is understood that a limitation of the S-system model is that the parameters may not be identifiable when the concentrations and reaction rates are very small. However, considering that the cell interior is homeostatic, this condition is unlikely to occur during the flowering time of *Arabidopsis*. Another possible difficulty with the S-system is the low sampling intervals of the genes required for parameter identification, which is also a challenge for all other kinetic models. For parameter estimation we assumed that all the 8 genes are measurable.

**Figure 3:**
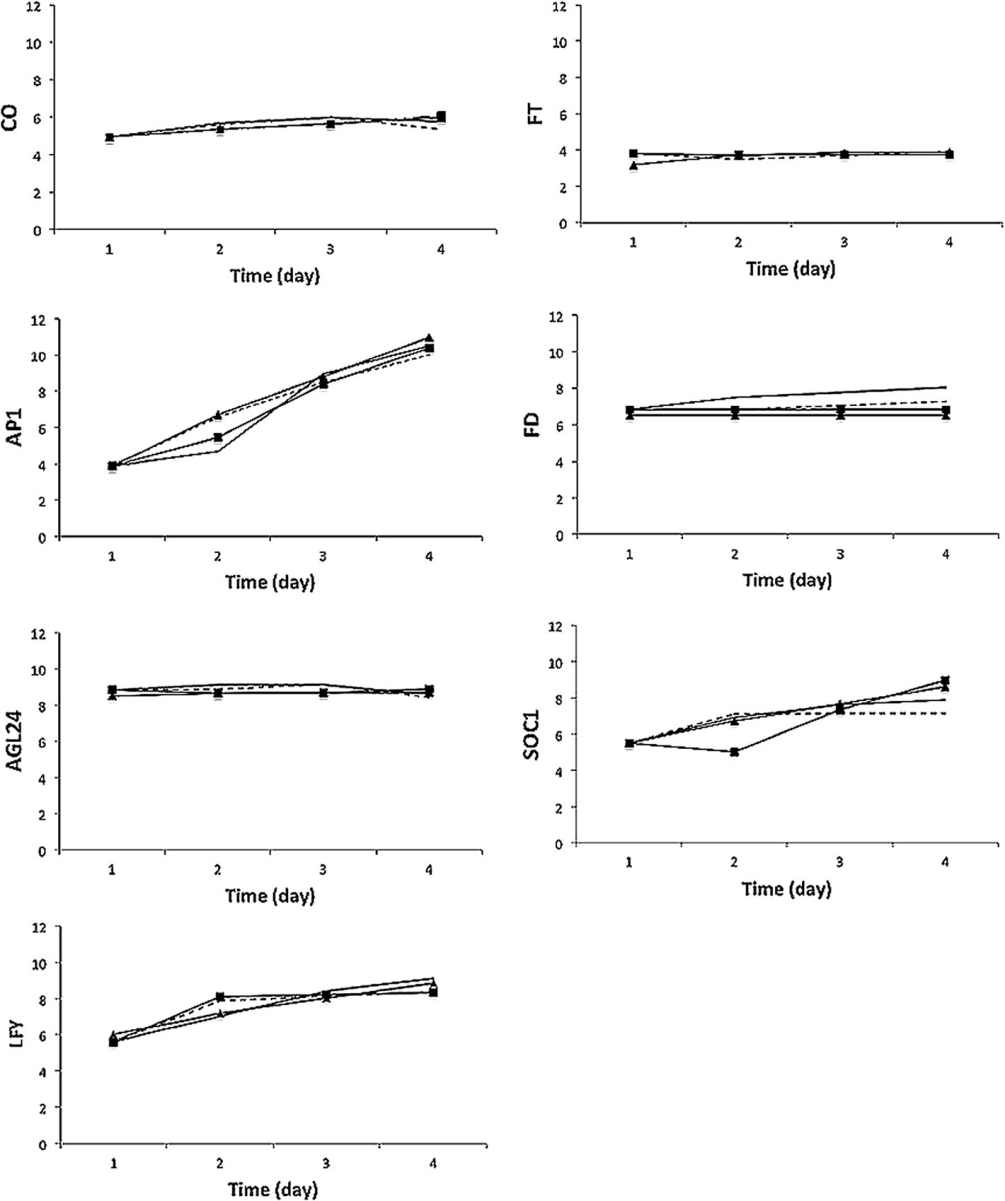
**A comparison of the simulated regulation of the flowering time in *Arabidopsis (0, 3, 5, and 7 day)CONSTANS (CO), FLOWERING LOCUS T (FT), Protein FD (FD), SUPPRESSOR OF CONSTANS OVEREXPRESSION 1 (SOC1), APETALA1 (AP1), AGL24 *** **and *LEAFY (LFY*) for the expression data of *ler *** **(black solid line) and other models (S-system ****, Mass-action model ****, Michaelis-Menten model ****).**

The estimated parameter values are listed in Additional file 1. Figure 3 shows the simulated time course data for the following genes: *CONSTANS (CO), FLOWERING LOCUS T (FT), protein FD (FD), SUPPRESSOR OF CONSTANS OVEREXPRESSION 1 (SOC1), APETALA1 (AP1), AGL24* and *LEAFY (LFY).* It is noted that the discrepancies between the S-system predicted values and the mRNA expression levels were relatively small for all of the modeled genes, suggesting that it may successfully replace the other two models to simulate time course data.

### Performance of the three models

In this study, we compared three alternative models: the S-system, Michaelis-Menten model, and Mass-action model, for the flowering time regulatory network of *Arabidopsis*. Both the Michaelis-Menten model and Mass-action model ignore the diffusion effect in the reaction. The Mass-action model assumes that the TF initiates mRNA transcription immediately, whereas the Michaelis-Menten model describes TF binds with DNA first and with the active mRNA later. The S-system models this process by adopting the 1D and 3D diffusion-reaction mechanisms. The 1D diffusion-reaction mechanism assumes the TF binds with the target site then activates the mRNA. The 3D diffusion-reaction mechanism describes that the TF binds with the DNA first then diffuses along the DNA to search for the target site, activating the mRNA transcription process during the final stage.

Shown in Figure 1 and Figure 2 are the *MRE* and *O(p)* calculated for the seven floral transition genes. The results indicate that the S-system, when compared with the real experimental data, achieved the best prediction.

### Sensitivity analysis of the models

Sensitivity analysis can be applied to estimate the effect of parameter changes, while MRE gives an estimate of a model’s rate of change based on local sensitivity analysis. In this section, we report the results of the time dependent sensitivity analysis [Eq. 8] for a time period of 100 seconds.

**Figure 4:**
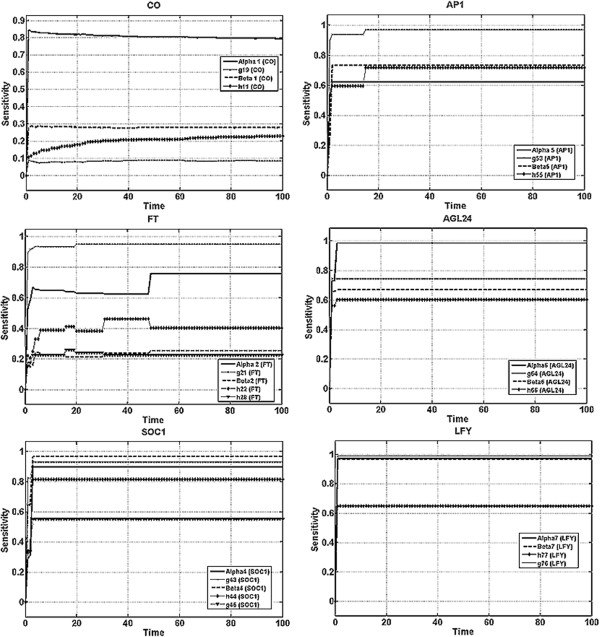
**Time-dependent sensitivity analysis of the parameters in the S-system, where *a *** **and *b *** **are system function parameter vectors (alpha and beta) consisting of rate constants, and *g *** **and *h *** **are kinetic orders for genes *CONSTANS (CO), FLOWERING LOCUS T (FT), Protein FD (FD), SUPPRESSOR OF CONSTANS OVEREXPRESSION 1 (SOC1), APETALA1 (AP1), AGL24 *** **and *LEAFY (LFY).***

As shown in Figure 4, it is obvious the sensitivity measure is positive for every gene. This implies that the mRNA concentration increases due to perturbation. The reason is that all of the interactions were activations. The results of the S-system show that fluctuations are limited to the first 10 seconds only, which is relative short compared to 50 seconds for the Michaelis-Menten model or mass action model (see Additional file 2 and Additional file 3). This in turn suggests that the response is a transient effect at most. A sensitivity value near zero means that gene activity is not sensitive to parameter perturbation at all. For a given gene, the response curves for the rate constants reflect the effect of perturbation on the transcription rate. For the kinetic order response, the sensitivity results indicate the effects on the strength of the activation or suppression.

Although an FT/FD complex formation measurement was not available, as long as we assumed a steady state approximation, i.e., [FD/FT] = k[FD][FT], the S-system was still capable of giving a reasonable fitting for the complex’s regulated genes: SOC1 and AP1.

In this study, we focused on the experiments for fitting the parameters. With the sensitivity analysis we identified the most sensitive parameters and sampling time intervals; this may provide some directions for future experimental design for model refinement.

## Discussions

Production of mRNA is dominated by the binding process between TF and its target gene. This binding process can be described by a diffusion-reaction mechanism. Although the Michaelis-Menten model has been widely used in gene regulation models, the results based on the MRE values show that the S-system is a better dynamic model for describing the flowering time of *Arabidopsis*. But the AIC values for the S-system (shown in Table 4) were larger than those of the Mass-action model, which implies an over-fitting may occur in parameter estimation.

The deviation is significant between the simulated data and experimental data for genes *AGL24* and *SOC1* in all the three models. These two genes form a feedback loop in the regulatory network; therefore such interactions degrade the performance of the three models. For a more complex regulation model, additional factors should be considered in the transcription mechanism. The values obtained from the sensitivity analysis were all positive, which indicate that the mRNA production rate is proportional to the collision frequency as well as the binding force between the TF and its target gene.

One may suggest that the performance effect is more due to the modeling technique rather than the equation system. This study only compared three possible models for one dynamic system. As the Mass-action model and Michaelis-Menten model techniques do not consider diffusion, they did not perform as well as the S-system. While the S-system seems to be most promising, a general conclusion that it is a better approach for modeling complex large-scale networks is yet to be established.

We used three reaction mechanisms to describe the process that a transcription factor binds on the promoter to generate mRNA. The Mass-action model assumes that this is a simple second-order reaction; the Michaelis-Menten model assumes that there is an intermediate product; and the S-system considers the diffusion effects in one dimension and three dimensions. Among the three, the Mass-action model is the simplest and has the least parameters. It is therefore less computation-intensive with the smallest AIC value (see Table 4). The disadvantage, however, is that it assumes the system is homogeneous which is not the case in our study. The Michaelis-Menten model also assumes the system is homogeneous and ignores the intracellular protein transport process. The S-system model does describe the diffusion effects which is the main driving force for mass transport in the cell. A disadvantage of the S-system is that it involves the most parameters. The largest AIC value of the S-system also implies an over-fitting may occur in parameter estimation.

We also considered the use of the Hill equation. In general the Hill equation is employed to describe the phenomenon that binding of a ligand to a macromolecule influencing the other ligands binding on the same macromolecule, which is known as cooperative binding. The Hill coefficient is used to quantify this effect, where a value of 1 indicates a completely independent binding, a value greater than 1 indicates a positive cooperativity, and a value less than 1 indicates a negative cooperativity. That is to say, a plurality of the same or different transcription factors are bound in the promoter region of a gene, and the first transcription factor affects the subsequent transcription factors in the promoter region. However, in our gene regulatory system, the number of transcription factors for the regulating genes is mostly one, and all the transcription factors have only one binding site on the promoter region. Therefore the Hill equation was not applicable in our study.

## Conclusions

One of the major problems of establishing large-scale dynamic models is the lack of experimental data. The parameters are usually unknown, so are the specific reaction rate laws. Moreover, for a large number of reactions, the parameters are only available in the literature whose values have to be obtained in *in vitro* conditions.

In this study, we focused on the molecular mechanism of transcription to propose models for describing the gene regulatory interactions of the flowering transition processes in *Arabidopsis*. The S-system has the best performance. Although we assumed that the best performance had come from the consideration of diffusion effects, its highest AIC values indicated a possible over-fitting in parameter estimation. It is therefore necessary to carefully apply the S-system for modeling complex gene regulatory networks. The diffusion effects may as well be included in the parameters for the Mass action model and the Michaelis-Menten model.

## Methods

### The regulation of flowering time network

The state transition to flowering in plants is precisely controlled by environmental conditions and endogenous developmental cues so that plants produce their progeny under favorable conditions. The response to multiple factors suggests the existence of a complex network regulating this state transition in plants. In this study, the biology of flowering time (Photoperiodic) in *Arabidopsis thaliana* showed that a complex gene regulatory network that controls this transition integrates the responses based on various external and internal conditions. Consequently, the regulation of the flowering time has been a major adaptive trait during plant evolution and domestication. A large number of genes have been characterized as flowering time regulators, and several recent reviews have provided detailed descriptions of flowering time pathways [2].

*Arabidopsis* is a facultative or quantitative long day plant that can flower, albeit much later in a short day. Key regulatory genes appear to be conserved in *Arabidopsis.* A short day plant suggests that common pathways are utilized [16,17]. The plant perceives photoperiod and is transduced to a downstream signaling system. The light-dependent pathway controls the flowering in response to seasonal changes.

**Figure 5:**
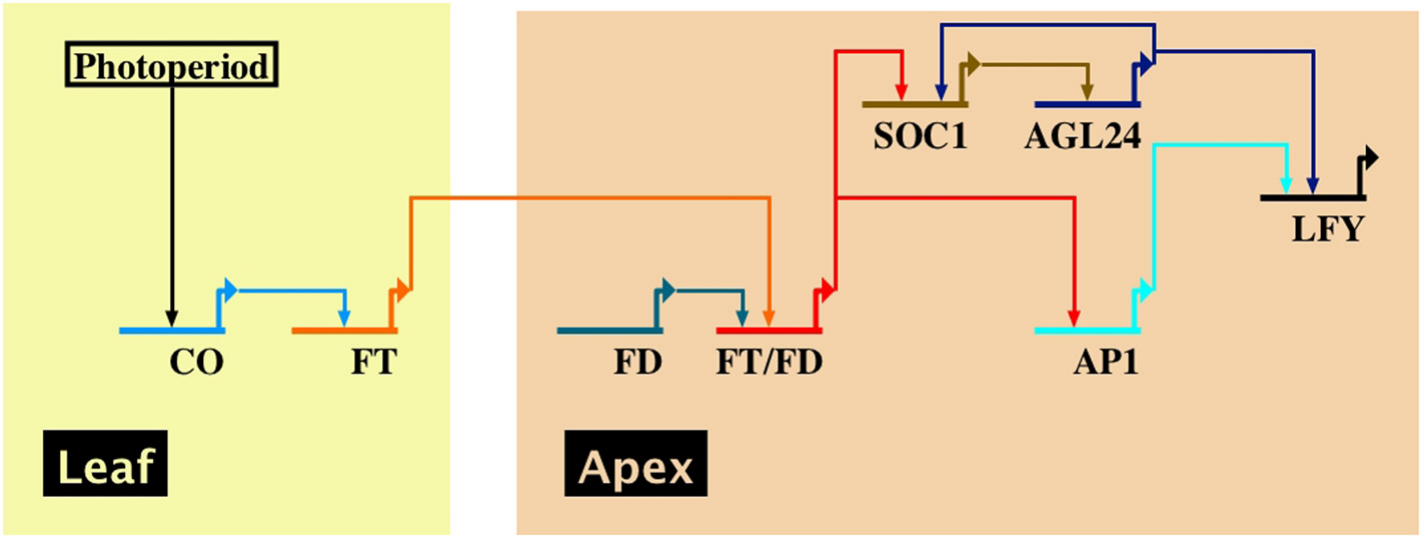
**Photoperiod pathway for the flowering transition process in *Arabidopsis*.**

Figure 5 shows the gene regulatory pathway for the flowering transition pathway in *Arabidopsis*[1], which is mediated by *CONSTANS* (*CO):*

1 *CO* codes for a zinc finger and *CCT* domain-containing transcription factor that accumulates under long day conditions in leaves as a result of the combination of the rhythmic expression of *CO* mRNA.

2 *CO* activates the expression of *FLOWERING LOCUST* (*FT*) probably by binding to the *FT* regulatory regions [18,19]. The FT protein is a component of the mobile flowering signal that moves upon its expression in the vascular tissue of leaves to the shoot apex [20,21].

3 At the meristem, *FT* physically interacts with the *bZIP* transcription factor *FD* and the *FT/FD* complex and activates the expression of *SOC1*[22].

4 *SOC1* and *AGL24* form a positive feedback loop, and the two factors might form a complex for the up-regulation of *LFY*. Thus, the regulators of the floral transition form a small network with multiple interactions among themselves,

5 *AP1* and *LFY* are ultimately resolved in the up-regulation of the floral meristem identity genes.

We used three different models for the flowering transition pathway in *Arabidopsis* to reconstruct the experimental data.

### Experimental data

We obtained the experimental data from:

(1) Plant Expression Database (http://www.plexdb.org/), ID: AT4;

(2) NCBI GEO, ID: GSE576 and GSE577.

Both contain the microarray data of the eight genes included in our study. Between the two, AT4 discusses the flower development of *Arabidopsis thaliana; it* was controlled by several signaling pathways which converge on a set of genes such as FT and SOC1 that function as pathway integrators. The photoperiod is regarded the most important factor in promoting floral transition: *Arabidopsis thaliana* will flower earlier under long day conditions than under short day conditions. It is therefore considered a facultative long day plant. To monitor changes of gene expression during floral transition and early flower development, plants were grown under short day (9 hr light, 15 hr dark) for 30 days and then shifted to long day (16 hr light, 8 hr dark) to induce flowering. The RNA was isolated from micro-dissected apical tissue harvested 0, 3, 5, and 7 days after hybridized to the Affymetrix ATH1 microarray.

We not only analyzed the reopens of *Columbia* (*col*) and *Landsberg erecta* (*ler*) but also the effect of mutants in the flowering time genes CONSTANS (*co*) and FT (*ft*). In this study, we used four experiments for parameter estimation: (1) the *Columbia* (*col*) is the most widely-used wild type of *Arabidopsis thaliana*; (2) The *Landsberg erecta* (*ler*) is currently the second most widely-used accession of *Arabidopsis thaliana*; finally (3) CONSTANS (*co*) and (4) FT (*ft*) are mutants in the flowering time [6].

We used four different experimental data sets based on optimized parameter estimation to find the most appropriate model.

### Dynamic model

Before introducing the transcription mechanism of the binding process, the following assumptions were made in advance: (a) Transcription is initiated when all the activation binding sites are occupied, and all the repression binding sites regarding the same gene are empty; and (b) The cell size remains constant during the time course of flowering state transition.

### S-system

**Figure 6:**
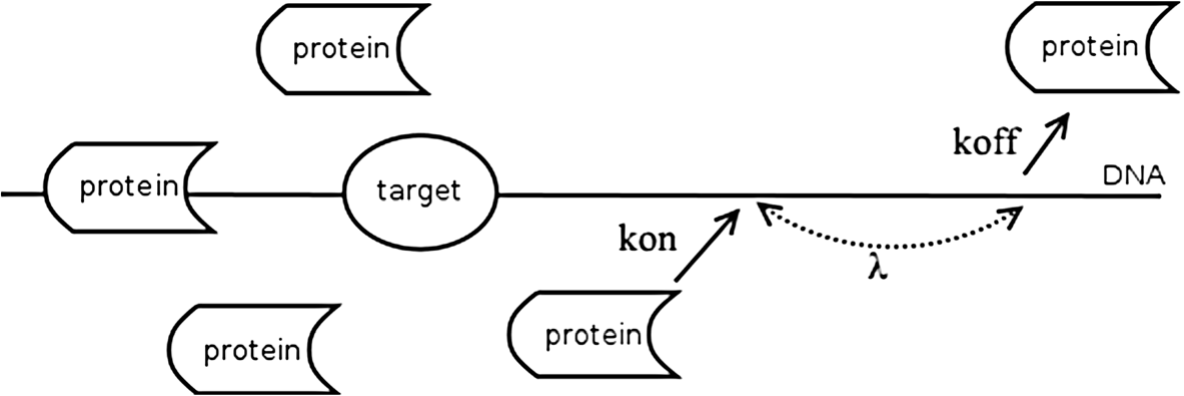
**Protein searches the target on DNA.** The kon and koff are adsorption and dissociation rate constants for protein and *λ* is the average length that each protein moves along DNA.

Most biological systems are nonlinear. Although the Michaelis-Menten model has been widely used to approach biological systems, one of the disadvantages lies in the fact that it is not an explicit mathematical form for all cases, especially for complex processes such as diffusion-reaction interactions. The S-system consists of a set of mathematical terms that is sufficient to capture most of the nonlinear phenomena in nature including diffusion-reaction interactions. The development of the S-system [23] has been shown to provide a good approximation for many cases, and there are efficient procedures for estimating the parameter values [24,25]. Protein-DNA interactions such as the binding between a transcription factor and its target gene have been studied for many years. Experimental observations have promoted the proposition that the diffusion effects in 3-D and 1-D crawled along the DNA are critical in the binding process. Early studies have yielded an unexpected result that the binding rate for the Lac repressor protein to its binding site on DNA is approximately 100-1000 times faster than the maximal 3-D diffusion rate in solution [26]. This phenomenon is called facilitated diffusion [27]. A picture of facilitated diffusion is schematically shown in Figure 6.

The process can be described by the reaction

$$\mathrm{TF}↔TF_{\mathrm{ns}}$$

$$TF_{\mathrm{ns}}+\mathrm{BS}\to \text{mRNA}$$

where *TF* represents the transcription factor, *TF*_*ns*_ represents the non-specific absorbed transcription factor on the DNA, and BS represents the binding site. The first step in this reaction is absorption of the transcription factor on the DNA. The second step in this reaction is mRNA production after the absorbed transcription factor has bound to its target gene. The mRNA production rate can be formulated as [28]:

(1) $$v=\frac{\lambda \left[TF_{\mathrm{ns}}\right]}{L\tau }$$

where λ is the average length of the transcription factor that moves along the DNA, *L* is the total length of the DNA, and τ is the sum of the 3-D diffusion time and the retention time on the DNA for the transcription factor.

The Langmuir isotherm is not suitable for describing protein adsorption, because the diverse conformations and multiple absorbed sites in the absorption process [29]. The Freundlich adsorption isotherm is more concordant with the experimental observations of proteins absorption. Proteins absorption is strongly dependent on the bulk concentration of proteins. In this case, the Freundlich adsorption isotherm of transcription factor on the DNA can be expressed as

(2) $$\left[TF_{\mathrm{ns}}\right]=K{\left[\mathrm{TF}\right]}^{1/n}$$

where K and n are constants at a particular temperature. From equations (1) and (2), the mRNA production rate can be determined as:

(3) $$v=\frac{\lambda k}{L\tau }{\left[\mathrm{TF}\right]}^{1/n}$$

It can be transformed to the mathematic form of the S-system. For this reason, we adopted the S-system function to describe the diffusion-reaction interactions in the mRNA transcription process. In the case of mRNA transcription, the S-system equation for the transcription rate is given by:

(4) $$v_{i}=\alpha_{i}\overset{n}{\underset{j=1}{\prod }}{\left[TF_{j}\right]}^{g_{{}^{\mathrm{ij}}}}-\beta_{i}\overset{n}{\underset{j=1}{\prod }}{\left[TF_{j}\right]}^{h_{{}^{\mathrm{ij}}}}$$

where *n* is the number of transcription factors, *TF*_*j*_ is the *j-th* transcription factor that regulates gene *i*, *α*_*i*_ and *β*_*i*_ denote the positive rate constants, and *g*_*ij*_ and *h*_*ij*_ are referred to as the kinetic orders. If *g*_*ij*_ > 0, gene *j* will induce the expression of gene *i*. On the contrary, gene *j* will inhibit the expression of gene *i* if *g*_*ij*_ < 0. The variable *h*_*ij*_ has the opposite effects in controlling the gene expression compared to *g*_*ij*_. In the present study, the range of *g*_*ij*_ and *h*_*ij*_ falls between 0 and 2.

### Michaelis–Menten model

The Michaelis-Menten model describes a catalysed reaction in which an intermediate complex forms after binding between enzyme and substrate. Thereafter, the complex (*TF-BS*) converts to the product and enzyme. In the case of mRNA transcription, the transcription process via such mechanism may be represented as:

TF+BS↔TF-BS

TF-BS→mRNA

Under the condition [TF]> > [BS], the production rate of mRNA for a gene with the diffusion effect ignored can be formulated as:

(5) $$v=\frac{V_{\text{max}}\left[\mathrm{TF}\right]}{K_{m}+\left[\mathrm{TF}\right]}$$

where *V*_*max*_ is the maximum production rate of mRNA and *K*_*m*_ is the Michaelis constant. The delay effect on the mRNA production increases with the stability of the complex state.

### Mass-action model

The chemical mechanism of Mass-action model states that the reaction rate is proportional to the product of the active mass of reactants. In the case of mRNA transcription, the reactants correspond to the transcription factor (TF) from the upstream gene and its specific binding site (BS) on the downstream gene. The transcription process in the cell may be represented as:

TF+BS→mRNA

Because the total number of binding sites for a specific gene is fixed, the production rate of mRNA of the downstream gene with diffusion ignored can be formulated as:

(6) $$v=k\left[\mathrm{TF}\right]$$

where *k* is the rate constant and [TF] represents the concentration of the transcription factor. The delay effect on the mRNA production is assumed to be zero.

### Parameter estimation

The objective of parameter estimation is to adjust the parameter values of a model via an optimization procedure so that the predictions based on the model can closely express the observation data. Parameter estimation can be performed through global methods and local methods [30]. However, one of the major challenges in modeling large-scale dynamic systems has been the existence of several local minima in the solution space. In this study, parameter estimation was performed by using the software tool “Complex Pathway Simulator” (COPASI Ver. 4.8) to fit the time series experimental data based on a dynamic model [31]. Evolutionary Programming (EP), Hooke & Jeeves (HJ) and Particle Swarm Optimization (PSO) were applied to search for an optimal solution, which may not converge to the minimum with different initial guesses. Among the three, EP [32,33] was inspired by biological evolution, PSO [34] was inspired by social behavior and the movement dynamics of insects, birds and fishes, and HJ [35] was derived based on a hill climbing technique.

These algorithms possess key advantages in large inverse problems of quantitative mathematical models [36]. The goodness of fitting for each set of estimated parameters can be quantified by the least squared error *O*:

(7) $$O\left(p\right)=\overset{n}{\underset{i=1}{\sum }}\overset{t}{\underset{j=1}{\sum }}\omega_{i}{\left(X_{i,j}-Y_{i,j}\left(p\right)\right)}^{2}$$

where *p* is the tested parameter set, *n* and *t* are the number of genes in the regulatory network and the number of samples in the time series data, respectively; *Y*_*i,j*_*(p*) is the prediction time series data by the dynamic model for the parameter set *p*; and *X*_*i,j*_ is the experimental time series data. The weighting factor is given by the mean square: $\omega_{i}=\frac{1}{\sqrt{\left(X_{1}^{2}\right)}}$.

### Model ranking and selection

In this study, we compared three models for the gene regulatory network of the flowering time regulation in *Arabidopsis*. We used the mean relative error (MRE) to quantify the response of each model subject to small perturbations [37]:

(8) $$\text{MRE}=\frac{\overset{n}{\underset{i=1}{\sum }}\left[\overset{t}{\underset{i=1}{\sum }}\left|\left(x_{i,j}-y_{i,j}\right)/x_{i,j}\right|\right]}{n}$$

where *x*_*i,j*_ denotes the experimental time series data for the *i-*th gene at time point *j, y*_*i,j*_ is the simulation data for the *i-*th gene given by the model at time point *j, n* is the total number of genes, and *t* is the number of samples in the time series data.

### Parameter sensitivity analysis

Dynamic models have been widely used to study metabolic networks and gene regulatory networks. These models are used to reconstruct experimental data and predict unobserved behaviors of a biological system. To address the many sources of uncertainty including error and noise in the experimental data, sensitivity analysis may be performed to identify the parameters in a model that have the strongest effect on the overall behavior.

Sensitivity analyses have the primary goal of determining how a given model responds to variations in a parameter. Local sensitivity analysis focuses on a particular point in the parameter space by changing one parameter at a time to obtain a local response of the model. Global sensitivity analysis tries to capture the entire parameter space all at once, allowing multiple parameters to be explored simultaneously [38].

In this study, we used the SBML-SAT software tool for Multi-Parametric Sensitivity analysis (MPSA) [39]. An MPSA analysis of the time dependent normalized sensitivity response is defined as:

(9) $$S_{\mathrm{ij}}\left(X_{j},p_{i}\right)=\frac{\partial \ln X_{j}}{\partial \ln p_{i}}$$

where *X*_*j*_is the mRNA concentration of the *j-*th gene, and *p*_*i*_ is the *i-*th parameter in the dynamic model.

### Akaike information criterion

We compared three models for flowering time regulation in *Arabidopsis*. We used several parameter estimation methods to estimate the parameters of the dynamic models.

Parameter Estimation helps us quantify the regulatory abilities of the genes involved at the flowering time. In order to determine whether a dynamic model is optimal, in this study a statistical approach called Akaike Information Criterion (AIC) [40] was employed to validate the number of model parameters and determine the significance of the parameters.

The Akaike Information Criterion (AIC), which attempts to include both the estimated residual variance and the model complexity in one statistic, decreases as the residual variance ***S***_***e***_ decreases, and it increases as the number of parameters *p* increases. For a gene regulatory model with *p* regulatory parameters to fit with a dataset of N samples, the Akaike Information Criterion (AIC) can be written as [40]:v

(10) $$\text{AIC}=2k-2\ln\left(L\right)$$

where ***L*** is the likelihood of the mathematical model and ***p*** is the number of parameters in the model.

## Competing interests

The authors declare that they have no competing interests.

## Authors’ contributions

CCNW, PCC and KLN: Mathematical model analysis, simulation studies, software development, data analysis and manipulation, and drafting of the article. CMC,PCYS, and JJPT: co-wrote the article. All authors read and approved the manuscript.

## Supplementary Material

Additional file 1 Identified parameters used in Particle Swarm Optimization (PSO) for S-system, Michaelis-Menten model and Mass action model. Click here for file

Additional file 2 The Michaelis–Menten model of time-dependent sensitivity analysis of parameters. Click here for file

Additional file 3 The Mass action model of time-dependent sensitivity analysis of parameters. Click here for file
