# Supplementary material for: A model comparison study of the flowering time regulatory network in Arabidopsis
Source: BMC Syst Biol. 2014 Feb 11;8:15. doi: 10.1186/1752-0509-8-15 (PMC3938817; doi:10.1186/1752-0509-8-15)
Supplement: Additional file 1 — Identified parameters used in Particle Swarm Optimization (PSO) for S-system, Michaelis-Menten model and Mass action model. [file 1752-0509-8-15-S1.docx]

## Additional 1 - Identified parameters used in Particle Swarm Optimization (PSO) for S-System, Michaelis-Menten model and Mass action model

Experimental data of *col*

| S-System, represents gene-speciﬁc synthesis constant, the gene-speciﬁc decay constant, the regulatory effects of other genes and the decay rate is determined by the regulatory effects of other genes. , , and rate in n*M* day^-1^ 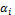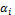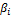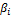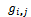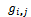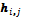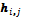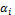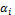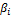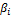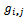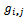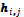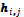 | | | | |  |
| --- | --- | --- | --- | --- | --- |
| 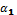 | 2.8475 | 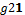 | | 3.0380 | |
| 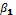 | 5.7564 | 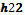 | | 4.9734 | |
| 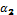 | 11.6048 | 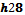 | | 0.2162 | |
| 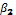 | 2.1992 | 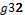 | | 1.1819e-11 | |
| 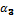 | 7.0377 | 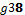 | | 1.3361 | |
| 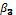 | 16.1553 | 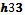 | | 3.4116 | |
| 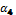 | 0.0409 | 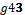 | | 4.9725 | |
| 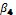 | 0.5290 | 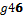 | | 0.2643 | |
| 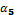 | 0.9790 | 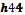 | | 0.0077 | |
| 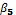 | 0.0001 | 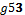 | | 0.0056 | |
| 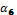 | 1.0404 | 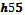 | | 0.3795 | |
| 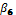 | 0.6815 | 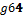 | | 1.3508 | |
| 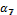 | 0.7323 | 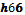 | | 1.3550 | |
| 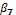 | 14.3765 | 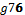 | | 2.6712 | |
| 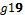 | 3.8821 | 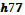 | | 1.4639 | |
| 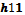 | 4.4552 |  | |  | |
| Michaelis-Menten model, represents the maximum transcription rate, the half-maximum activation or repression rate and the decay rate., and rate in n*M* day^-1^ 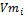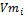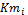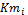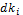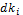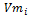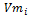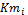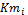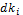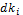 | | | | |  |
| 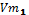 | 0.385841 | 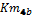 | 7.39321 | |  |
| 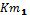 | 0.00346169 | 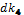 | 1.39424 | |  |
| 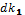 | 2.75379e-09 | 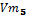 | 20 | |  |
| 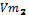 | 15.8787 | 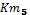 | 19.9874 | |  |
| 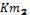 | 0.0001 | 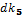 | 0.30201 | |  |
| 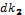 | 4.20066 | 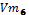 | 5.77678 | |  |
| 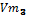 | 3.17216 | 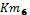 | 0.000207456 | |  |
| 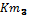 | 19.9984 | 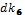 | 0.636979 | |  |
| 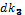 | 1.35716e-08 | 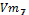 | 1.59943 | |  |
| 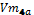 | 1.08712 | 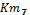 | 19.9996 | |  |
| 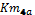 | 0.588139 | 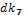 | 8.0208e-09 | |  |
| 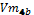 | 19.9913 |  |  | |  |
| Mass action model, represents the synthesis constant and the decay rate. and rate in n*M* day^-1^ 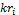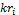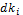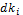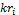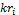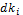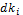 | | | | |  |
| 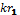 | 4.24919 | 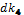 | 12.3477 | |  |
| 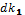 | 6.58466 | 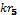 | 8.65925 | |  |
| 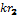 | 10.6094 | 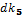 | 11.9552 | |  |
| 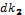 | 16.5102 | 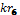 | 3.16427 | |  |
| 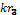 | 5.09683 | 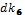 | 2.30839 | |  |
|  | 15.8639 |  | 15.8106 | |  |
|  | 0.198334 |  | 19.9908 | |  |
|  | 5.80586 |  |  | |  |

Experimental data of *ler*

| S-System, represents gene-speciﬁc synthesis constant, the gene-speciﬁc decay constant, the regulatory effects of other genes and the decay rate is determined by the regulatory effects of other genes., , and rate in n*M* day^-1^ | | | |
| --- | --- | --- | --- |
|  | 3.34204 |  | 1.13828 |
|  | 2.31608 |  | 3.76208 |
|  | 10.4078 |  | 0.154451 |
|  | 0.357435 |  | 0.0971682 |
|  | 0.383004 |  | 0.015866 |
|  | 0.348923 |  | 5 |
|  | 0.0211516 |  | 0.497373 |
|  | 0.0681673 |  | 2.64669 |
|  | 7.8235 |  | 2.24612 |
|  | 0.000878022 |  | 5 |
|  | 3.67971 |  | 3.94653 |
|  | 0.290635 |  | 1.93773e-013 |
|  | 0.0352574 |  | 1.14858 |
|  | 0.0001 |  | 1.38629 |
|  | 0.141247 |  | 4.98301 |
|  | 0.366292 |  |  |
| Michaelis-Menten model, represents the maximum transcription rate, the half-maximum activation or repression rate and the decay rate. , and rate in n*M* day^-1^ | | | |
|  | 0.457039 |  | 0.00021962 |
|  | 6.62869 |  | 9.55977e-009 |
|  | 1.2797e-009 |  | 19.9997 |
|  | 12.8068 |  | 3.71465 |
|  | 0.521998 |  | 1.78306e-006 |
|  | 3.06685 |  | 0.137496 |
|  | 0.367952 |  | 8.96663e-005 |
|  | 15.9053 |  | 6.671e-007 |
|  | 0.197215 |  | 2.36354 |
|  | 2.04805 |  | 19.9995 |
|  | 20 |  | 1.2419e-007 |
|  | 19.9995 |  |  |
| Mass action model, represents the synthesis constant and the decay rate. and rate in n*M* day^-1^ | | | |
|  | 0.345126 |  | 1.39504e-006 |
|  | 0.534698 |  | 18.234 |
|  | 12.9357 |  | 2.21234e-009 |
|  | 19.0632 |  | 1.07734 |
|  | 0.00302373 |  | 0.762982 |
|  | 0.426625 |  | 17.0164 |
|  | 0.0468619 |  | 19.9999 |
|  | 17.8823 |  |  |

By experimental data of *co*

| S-System, represents gene-speciﬁc synthesis constant, the gene-speciﬁc decay constant, the regulatory effects of other genes and the decay rate is determined by the regulatory effects of other genes., , and rate in n*M* day^-1^ | | | |
| --- | --- | --- | --- |
|  | 9.38793 |  | 0.916076 |
|  | 8.63877 |  | 1.04172 |
|  | 7.443 |  | 2.54345e-006 |
|  | 9.10962 |  | 2.58824 |
|  | 0.383001 |  | 2.03245 |
|  | 1.78665 |  | 2.55825 |
|  | 2.58161 |  | 3.97278 |
|  | 9.65942 |  | 0.0613727 |
|  | 8.97467 |  | 4.62128 |
|  | 1.34566 |  | 0.699766 |
|  | 0.263984 |  | 2.44607 |
|  | 2.33779 |  | 4.89369 |
|  | 0.0826941 |  | 2.94424 |
|  | 0.643899 |  | 1.7798 |
|  | 2.98809 |  | 1.03813 |
|  | 3.95792 |  |  |
| Michaelis-Menten model, represents the maximum transcription rate, the half-maximum activation or repression rate and the decay rate., and rate in n*M* day^-1^ | | | |
|  | 1.22837 |  | 0.00040037 |
|  | 20 |  | 1.04159 |
|  | 1.3313e-006 |  | 20 |
|  | 12.5908 |  | 14.711 |
|  | 2.37955e-005 |  | 0.301769 |
|  | 3.32704 |  | 0.148931 |
|  | 2.2026 |  | 0.00124483 |
|  | 17.33 |  | 0.0001 |
|  | 1.46997e-006 |  | 1.40702 |
|  | 4.17017 |  | 16.3977 |
|  | 0.441013 |  | 7.77037e-006 |
|  | 2.18607 |  |  |
| Mass action model, represents the synthesis constant and the decay rate. and rate in n*M* day^-1^ | | | |
|  | 0.343593 |  | 0.0001 |
|  | 0.496442 |  | 0.334729 |
|  | 11.7818 |  | 20 |
|  | 18.2643 |  | 0.00497383 |
|  | 6.56497 |  | 0.0001 |
|  | 0.0935268 |  | 0.0699055 |
|  | 14.543 |  | 0.0001 |
|  | 1.72368e-005 |  |  |

By experimental data of *ft*

| S-System, represents gene-speciﬁc synthesis constant, the gene-speciﬁc decay constant, the regulatory effects of other genes and the decay rate is determined by the regulatory effects of other genes., , and rate in n*M* day^-1^ | | | |
| --- | --- | --- | --- |
|  | 0.0180706 |  | 4.04168 |
|  | 6.8603e-005 |  | 4.4347 |
|  | 16.935 |  | 0.615724 |
|  | 14.2806 |  | 1.6458 |
|  | 5.35473 |  | 3.13208 |
|  | 19.8082 |  | 4.3588 |
|  | 1.3102 |  | 3.60404 |
|  | 16.8151 |  | 1.0391 |
|  | 8.24556 |  | 3.15949 |
|  | 0.570711 |  | 1.0011 |
|  | 1.22572 |  | 2.99265 |
|  | 0.62518 |  | 0.0186322 |
|  | 0.259111 |  | 0.238396 |
|  | 19.9769 |  | 2.69121 |
|  | 1.04836 |  | 0.867747 |
|  | 0.164051 |  |  |
| Michaelis-Menten model, represents the maximum transcription rate, the half-maximum activation or repression rate and the decay rate., and rate in n*M* day^-1^ | | | |
|  | 0.480894 |  | 1.05181 |
|  | 14.7807 |  | 0.0442232 |
|  | 1.57479e-007 |  | 15.9776 |
|  | 5.21509 |  | 14.6398 |
|  | 0.0001 |  | 3.94706e-008 |
|  | 1.39994 |  | 0.192337 |
|  | 0.111155 |  | 2.26689e-006 |
|  | 17.0804 |  | 2.4985e-006 |
|  | 1.54089e-008 |  | 0.401852 |
|  | 4.08459 |  | 20 |
|  | 19.9924 |  | 2.05324e-006 |
|  | 19.9941 |  |  |
| Mass action model, represents the synthesis constant and the decay rate. and rate in n*M* day^-1^ | | | |
|  | 6.77662 |  | 0.0001 |
|  | 11.7155 |  | 0.0001 |
|  | 11.0801 |  | 0.0001 |
|  | 16.1096 |  | 15.7285 |
|  | 0.0001 |  | 9.58868 |
|  | 7.51923 |  | 12.5122 |
|  | 1.73293 |  | 18.3915 |
|  | 4.60061 |  |  |
