# Supplementary figures and images for: A model comparison study of the flowering time regulatory network in Arabidopsis
Source: BMC Syst Biol. 2014 Feb 11;8:15. doi: 10.1186/1752-0509-8-15 (PMC3938817; doi:10.1186/1752-0509-8-15)

## Additional 2 - The Michaelis–Menten model of time-dependent sensitivity analysis of parameters


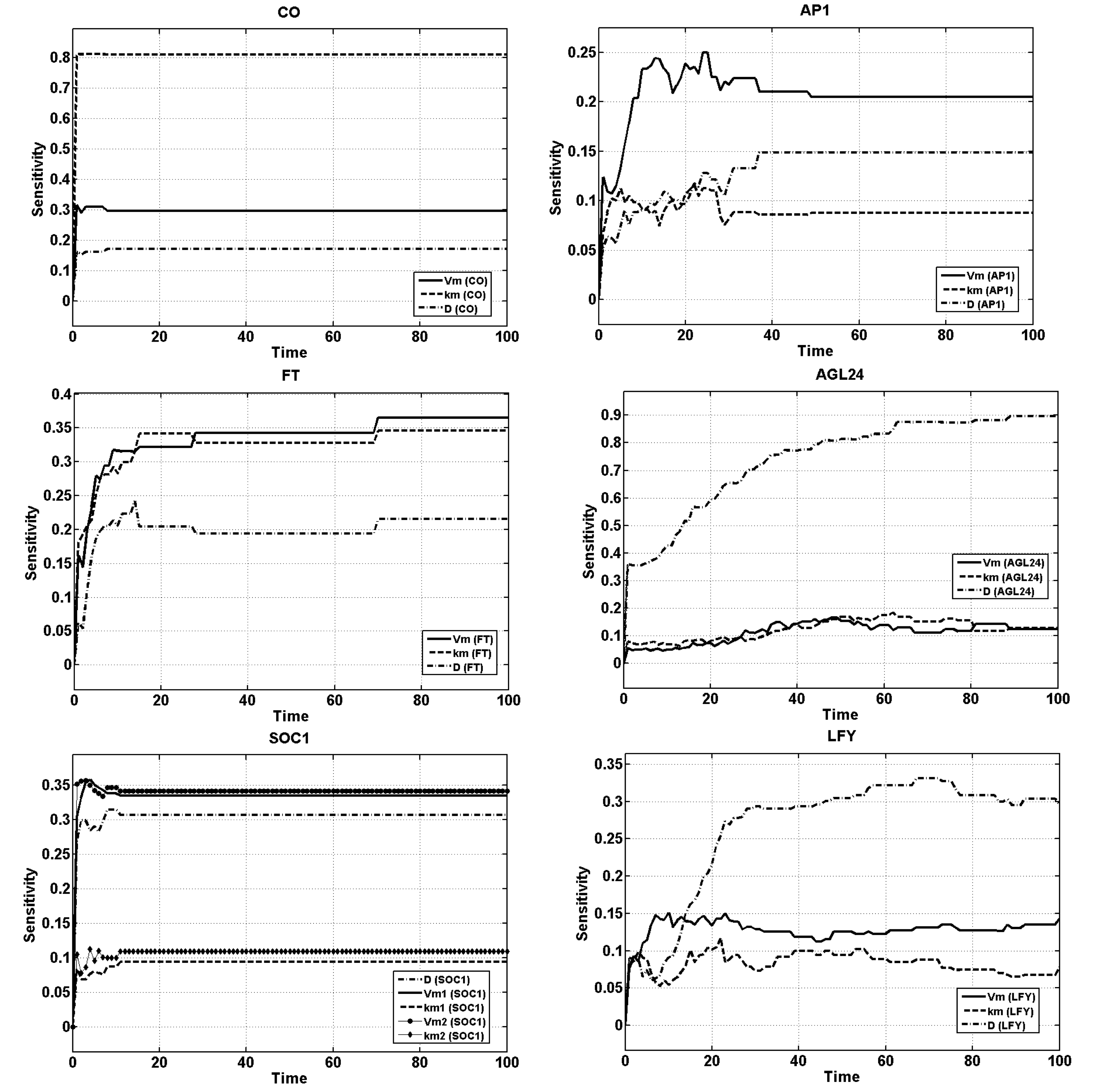

Supplement: Additional file 2 — The Michaelis–Menten model of time-dependent sensitivity analysis of parameters. [file 1752-0509-8-15-S2.docx]

## Additional 3 - The Mass action model of time-dependent sensitivity analysis of parameters


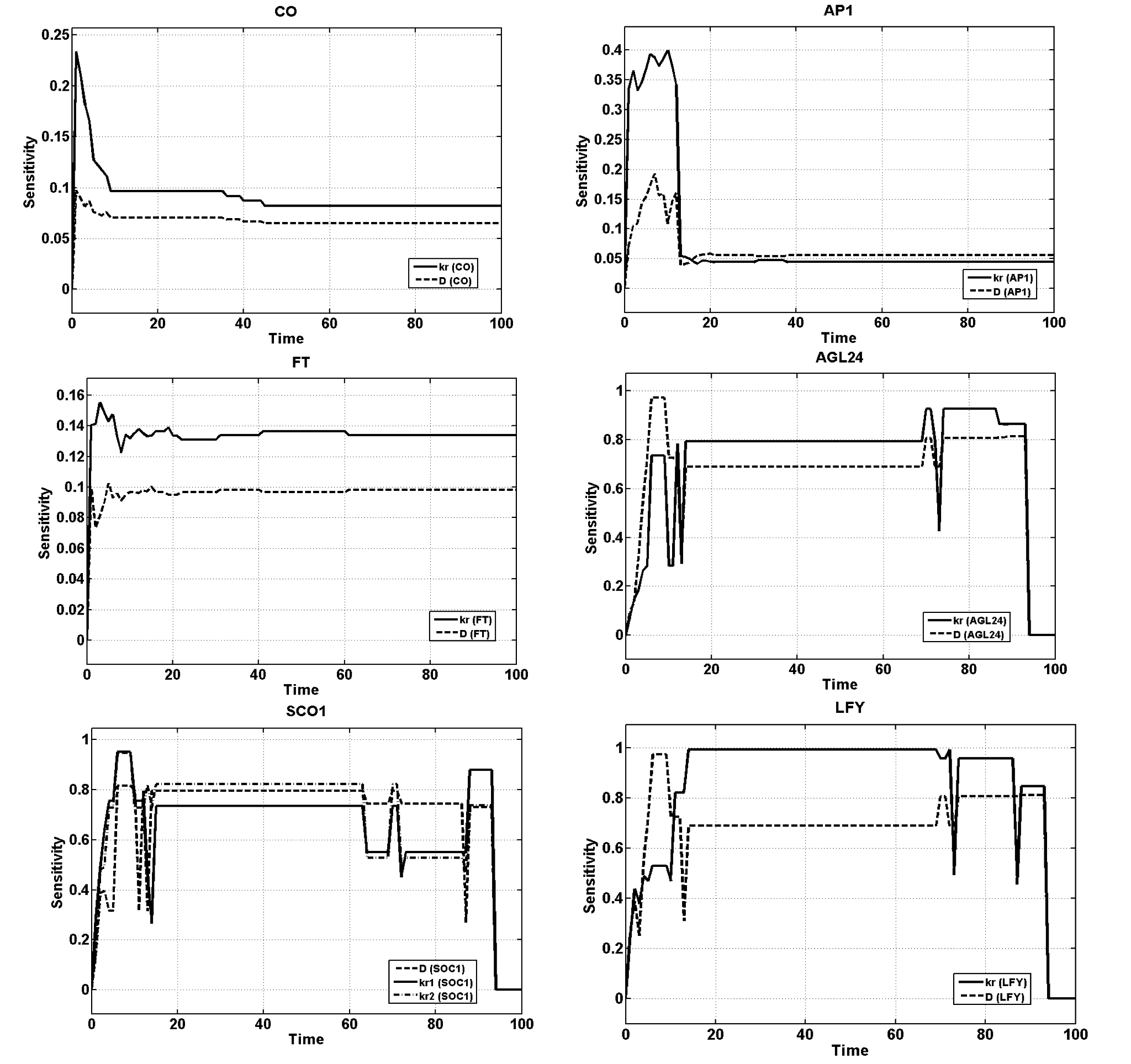

Supplement: Additional file 3 — The Mass action model of time-dependent sensitivity analysis of parameters. [file 1752-0509-8-15-S3.docx]
